# Pelvic lipomatosis—a rare diagnosis and a challenging management: a case report and literature review

**DOI:** 10.1093/jscr/rjae777

**Published:** 2024-12-15

**Authors:** Yaser M Ata, Fatima A Al-Jassim, Kholoud Alabassi, Ahmed Albakr, Taha Ismail, Khalid Al Jalham

**Affiliations:** Urology Department, Hamad Medical Corporation, Doha, Qatar; Urology Department, Hamad Medical Corporation, Doha, Qatar; Urology Department, Hamad Medical Corporation, Doha, Qatar; Urology Department, Hamad Medical Corporation, Doha, Qatar; Urology Department, Hamad Medical Corporation, Doha, Qatar; Urology Department, Hamad Medical Corporation, Doha, Qatar

**Keywords:** pelvic lipomatosis, LUTS, urinary diversion

## Abstract

Pelvic lipomatosis (PL) involves nonmalignant excess fat growth in perirectal and perivesical areas, gaining recognition over three decades. This review addresses its clinicopathological aspects amid controversies, emphasizing the need for a comprehensive examination of current literature. This report outlines a 42-year-old man’s diagnostic journey for persistent urological symptoms, ultimately identified as PL. PL, if untreated, adversely impacts the urinary system, affecting kidney function and causing systemic complications. Management involves accurate diagnosis through radiological methods and surgical intervention, aiming to alleviate symptoms and enhance affected individuals’ quality of life.

## Introduction

Pelvic lipomatosis (PL) is a rare benign condition marked by diffuse fatty tissue growth around pelvic organs [1]. First described by Engles in 1959 [2] and named by Fogg and Smyth in 1968 [3], PL affects 0.6–1.7 per 100 000 hospital admissions [3], with an average presentation age of 48. It is more common in males (male-to-female ratio 1.8:1) and has a higher prevalence in African Americans compared to Caucasians [4]. The exact cause is unknown, but it is often linked to obesity, with 50% of patients being obese [5, 6], and may have a hereditary component related to high mobility group A (HMGA) protein abnormalities [7].

Diagnosing PL is challenging due to its rarity and requires a combination of clinical and investigative data. Symptoms vary from pelvic compression, with flank or lower abdominal pain being most common [8]. Other symptoms include lower urinary tract symptoms (LUTS), hematuria, urinary stone symptoms, painful ejaculation, constipation, tenesmus, rectal bleeding, lower limb edema, and deep venous thrombosis (DVT) symptoms [8–10]. No specific lab markers exist for PL, though urinary tract obstruction can impact renal function tests.

Imaging is essential for diagnosing PL. Plain abdominal radiographs may reveal increased lucency in perivesical areas, and intravenous urograms can show bladder compression and elevation due to pelvic fat. Barium enema may display sigmoid colon and rectum compression [10]. Contrast-enhanced CT, with a sensitivity of 40.6% and specificity of 100%, is the most reliable method for PL [11, 12]. CT can show bladder shape changes (e.g. gourd or pear-like) and sigmoid compression [12, 13]. Magnetic resonance imaging (MRI) is less preferred due to its lower fat contrast [14].

Differential diagnoses for PL include lipoma, pelvic teratoma, retroperitoneal fibrosis, and liposarcoma [15]. Histologically, PL appears as dense, vascular lipomatous tissue, unlike simple lipomas. About 70% show mature cells, while 30% exhibit inflammation and vascular proliferation [4].

We report a 42-year-old man with PL causing LUTS, bilateral ureteral obstruction, urinary diversion, and ureteral reimplantation. Progressive PL led to severe bladder and urinary tract dysfunction.

## Case presentation

A 42-year-old Egyptian man with no co-morbidities presented with a bladder mass detected on ultrasound at a private facility for lower urinary tract symptoms. He experienced moderate voiding and storage symptoms but had no hematuria, and his clinical exam and lab results were within normal limits.

Flexible cystoscopy revealed a clear urethra and prostate but showed granulomatous reaction and bullous edema at the bladder neck and trigone, with the ureteric orifices not visible. Urine cytology was negative for high-grade malignancy. Cystoscopy with transurethral resection of the bladder neck and trigonal lesions was performed. Post-resection, the ureteric orifices were visible, and histopathology confirmed florid cystitis glandularis without malignancy.

During follow-up, the patient’s creatinine levels rose, and hydronephrosis worsened. Despite normal lower urinary tract symptoms and urine flowmetry (maximum flow rate 22 ml/s, post-void residual 5 ml), a renogram showed left kidney function at 85% and right at 15%, indicating obstructive uropathy. A right percutaneous nephrostomy revealed severe hydroureteronephrosis with obstruction at the lower ureter. Failed antegrade stenting necessitated abdominal exploration, which uncovered PL compressing both ureters. Bilateral ureterovesical reimplantation was performed.

**Figure 1:**
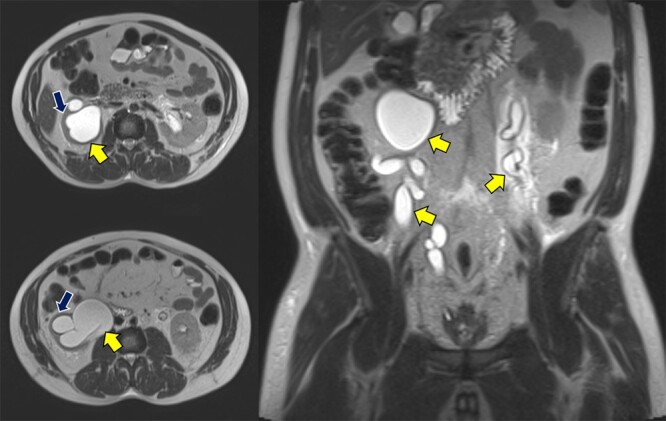
Abdominopelvic MRI T2WI demonstrating severe bilateral hydroureteronephrosis (yellow arrows) with thinning of the right renal cortex (blue arrow).

Abdominopelvic MRI T2-weighted imaging demonstrated severe bilateral hydroureteronephrosis (Fig. 1, yellow arrows) with thinning of the right renal cortex (blue arrow), suggestive of chronic obstruction.

**Figure 2:**
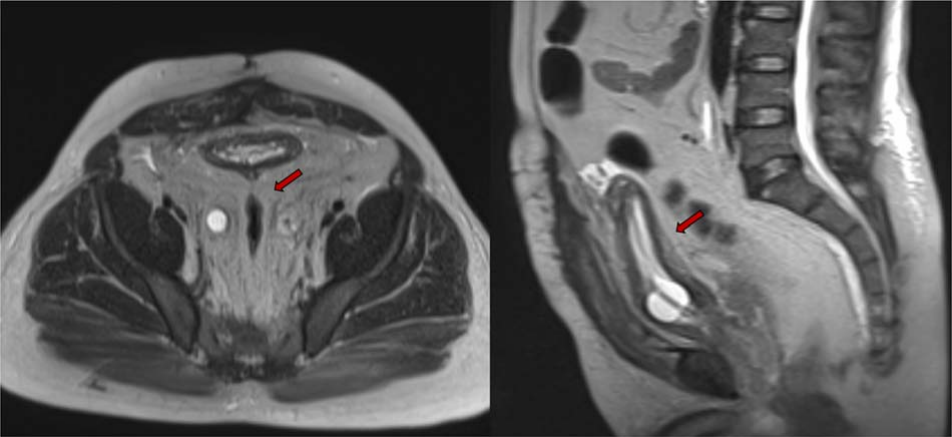
Abdominopelvic MRI T2WI PL surrounding urinary bladder and rectum (red arrow).

**Figure 3:**
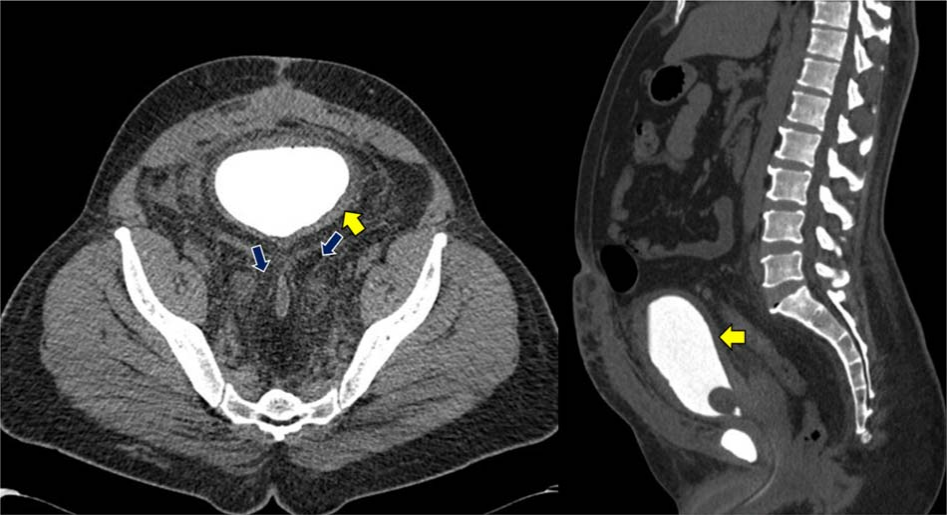
Axial and sagittal CT cystogram demonstrating urinary bladder wall thickening (yellow arrow) with PL and extensive fat strandings (blue arrows).

Despite initial stability, the patient’s creatinine levels rose again, and hydronephrosis worsened. Left percutaneous nephrostomy and antegrade stenting ultimately stabilized his creatinine, and he now undergoes annual stent exchanges. Follow-up abdominopelvic MRI T2WI revealed PL surrounding the urinary bladder and rectum (Fig. 2, red arrow). Additional imaging with axial and sagittal CT cystogram showed urinary bladder wall thickening (Fig. 3, yellow arrow) accompanied by extensive PL and fat stranding (blue arrows).

## Discussion

PL involves extensive benign fibro-adipose tissue with inflammatory response in the perivesical and perirenal spaces [13]. Our review of PL literature found only scattered case reports and a few small series with fewer than 10 cases.

PL can present with a range of symptoms due to compression of pelvic structures [9]. The most common symptom is flank or lower abdominal pain [8], while other symptoms include LUTS, hematuria, urinary stone symptoms, painful ejaculation, constipation, tenesmus, rectal bleeding, lower limb edema, and deep vein thrombosis symptoms [8–10]. Our patient had LUTS due to bladder compression by pelvic fat and inflammation. Although flank pain is common in PL [4], our patient did not experience it, likely due to chronic silent obstructive uropathy causing severe bilateral hydronephrosis.

PL is very similar to retroperitoneal fibrosis (RF) in that they are both characterized by abnormal tissue growth but differ in tissue composition and affected regions. Both conditions cause compressive symptoms, but RF commonly affects the ureters and abdominal organs, leading to obstructive uropathy, while PL primarily impacts the bladder and surrounding pelvic organs. Management and prognosis differ significantly; RF often requires immunosuppressive therapy, while PL management focuses on surgical interventions and symptom control [16].

PL can cause systemic effects such as secondary hypertension, deep venous thrombosis, and pulmonary embolism due to pelvic vascular compression [4]. Hypertension occurs in 35%–75% of PL patients [17]. Our patient was hypertensive and on treatment, consistent with literature.

Investigations reveal that plain abdominal radiographs can show increased lucency in perivesical areas [18]. Intravenous urograms may show bladder compression and elevation by pelvic fat, while barium enema can reveal sigmoid colon and rectum compression [10]. Contrast-enhanced CT is highly specific for bladder shape deformity in PL, though its sensitivity is low [12, 19, 20]. Bladder shapes can vary (e.g. gourd-like, pear-shaped) [12, 14]. CT can also show simultaneous sigmoid colon compression by pelvic fat. MRI can identify pelvic fat with T1 and T2 hyperintensity and fat suppression sequences, and is comparable to CT for PL diagnosis, with better delineation of pelvic structures [13]. In our case, diagnosis was based on extensive fat compression seen on CT and MRI.

Our patient required multiple surgical interventions, including cystoscopy, transurethral resection, bilateral ureteral re-implantation, percutaneous nephrostomy (PCN) urinary diversion, and indwelling ureteral stents. In managing PL, surgical transposition of the ureters intraperitoneally is viable to prevent compression by adipose tissue. Ureteral reimplantation effectively relieves obstruction and improves renal function [21]. Laparoscopic bilateral ureteral reimplantation has shown significant symptom relief and reduced hydronephrosis. A modified ileal conduit also improved renal function postoperatively [22]. Postoperative use of ureteral stents may be needed to maintain patency, though not all patients require them after reimplantation [16].

PL is associated with cystitis glandularis in 80% of cases [11], as seen in our patient. No single surgery cures PL [23], but urinary diversion can improve quality of life [4, 23]. Surgical fat removal is ineffective [4, 17], and non-surgical treatments like antimicrobials, steroids, and radiation have limited value [4]. Renal function monitoring is essential, though no specific follow-up schedule exists; we check our patient’s kidney function every 3 months and perform ultrasounds every 6 months.

Histologically, PL is dense, vascular, and lipomatous without a capsule, covering the pelvic organs [4]. Differential diagnoses include lipoma, pelvic teratoma, retroperitoneal fibrosis, and liposarcoma [13]. Unlike simple lipomas, PL lacks a capsule and shows irregular spread, with 70% of cases having mature cells and 30% showing inflammation and vascular proliferation [4]. Our patient had prior open surgery for pelvic fat removal, though no histological evaluation was done.

## Conclusion

PL is a rare condition that can significantly impact the urinary system if unmanaged, leading to kidney function disruption and systemic complications. Effective management requires accurate diagnosis via radiological methods and surgical intervention to alleviate symptoms and improve quality of life.

## References

[1] Gupta SK, Singh M, Kumar V, et al. Pelvic lipomatosis: a rare case with a good surgical outcome. UroToday Int J 2012;5:art 4. 10.3834/uij.1944-5784.2012.04.04.

[2] Engels EP . Sigmoid colon and urinary bladder in high fixation: roentgen changes simulating pelvic tumor. Radiology 1959;72:419–22. 10.1148/72.3.419. 13634406

[3] Fogg LB, Smyth JW. Pelvic lipomatosis: a condition simulating pelvic neoplasm. Radiology 1968;90:558–64. 10.1148/90.3.558. 5642294

[4] Heyns CF . Pelvic lipomatosis: a review of its diagnosis and management. J Urol 1991;146:267–73. 10.1016/s0022-5347(17)37767-4. 1856914

[5] Carpenter AA . Pelvic lipomatosis: successful surgical treatment. J Urol 1973;110:397–9. 10.1016/S0022-5347(17)60231-3. 4742176

[6] Morettin LB, Wilson M. Pelvic lipomatosis. Am J Roentgenol Radium Ther Nucl Med 1971;113:181–4. 10.2214/ajr.113.1.181. 5096819

[7] Fedele M, Battista S, Manfioletti G, et al. Role of the high mobility group a proteins in human lipomas. Carcinogenesis 2001;22:1583–91. 10.1093/carcin/22.10.1583. 11576996

[8] Buitrago Sivianes S, Tallada Bunuel M, Vicente Prados FJ, et al. Pelvic lipomatosis. Diagnostic and therapeutic considerations apropos of 3 cases. Arch Esp Urol 2002;55:900–6. 12455280

[9] Hermie I, Hermie L, Coenegrachts K. Pelvic lipomatosis causing renal failure. J Belg Soc Radiol 2016;100:55. 10.5334/jbr-btr.1072. 30151461 PMC6100427

[10] Blau JS, Janson KL. Pelvic lipomatosis: consideration of the urinary tract complications. Arch Surg 1972;105:498–500. 10.1001/archsurg.1972.04180090101024. 5056947

[11] Heyns CF, De Kock ML, Kirsten PH, et al. Pelvic lipomatosis associated with cystitis glandularis and adenocarcinoma of the bladder. J Urol 1991;145:364–6. 10.1016/S0022-5347(17)38342-8. 1988733

[12] Zhang Y, Wu S, Xi Z, et al. Measuring diagnostic accuracy of imaging parameters in pelvic lipomatosis. Eur J Radiol 2012;81:3107–14. 10.1016/j.ejrad.2012.05.031. 22749803

[13] Klein FA, Smith MJ, Kasenetz I. Pelvic lipomatosis: 35-year experience. J Urol 1988;139:998–1001. 10.1016/S0022-5347(17)42744-3. 3361678

[14] SanjayPrakash J, Mathisekaran T, Jain N, et al. Robotic management of pelvic lipomatosis-experience with difficulties encountered and the techniques to successful outcomes. Eur Urol Open Sci 2020;21:33–40. 10.1016/j.euros.2020.08.004. 34337466 PMC8317900

[15] Miglani U, Sinha T, Gupta SK, et al. Rare etiology of obstructive uropathy: pelvic lipomatosis. Urol Int 2010;84:239–41. 10.1159/000277606. 20215833

[16] Fuochi C, Taddei L, Taddei G, et al. Lipomatosi pelvica e fibrosi retroperitoneale: due fasi di una medesima malattia? Considerazioni su tre osservazioni personali e revisione della letteratura [pelvic lipomatosis and retroperitoneal fibrosis: 2 phases of the same disease? Report of 3 cases and review of the literature]. Radiol Med 1982;68:633–42. Italian. 7146504

[17] Michajlowski J, Matuszewski M, Klacz J, et al. Acute urinary retention in a patient with extended cystitis glandularis. Cent European J Urol 2011;64:94–6. 10.5173/ceju.2011.02.art11. PMC3921714 24578874

[18] Mahlin MS, Dovitz BW. Perivesical lipomatosis. J Urol 1968;100:720–2. 10.1016/S0022-5347(17)62607-7. 5748603

[19] Gerson ES, Gerzof SG, Robbins AH. CT confirmation of pelvic lipomatosis: two cases. AJR Am J Roentgenol 1977;129:338–40. 10.2214/ajr.129.2.338. 409176

[20] Yesilkaya Y, Duymus M, Topcuoglu M. Pelvic lipomatosis: US and CT diagnosis. Biomed Imaging Interv J 2012;8:e12. 10.2349/biij.8.2.e12. 22970068 PMC3432258

[21] Zong D, Xu X, Yan K, et al. Ureteral reimplantation for the management of pelvic lipomatosis. IJU Case Rep 2024;7:181–4. 10.1002/iju5.12698. 38440700 PMC10909137

[22] Xia M, Meng C, Zhang P, et al. Modified ileal conduit for pelvic lipomatosis: technique description and outcome. Urol Int 2024;108:314–21. 10.1159/000538369. 38513631 PMC11305515

[23] Ge L, Tian X, Zhao G, et al. Surgical treatment for pelvic lipomatosis using a bladder-sparing technique: a STROBE-compliant study. Medicine (Baltimore) 2019;98:e16198. 10.1097/MD.0000000000016198. 31261563 PMC6616873

